# Entropic Origin of Ionic Interactions in Polar Solvents

**DOI:** 10.1021/acs.jpcb.3c00588

**Published:** 2023-05-09

**Authors:** Samuel Varner, Christopher Balzer, Zhen-Gang Wang

**Affiliations:** † Division of Chemistry and Chemical Engineering, California Institute of Technology, Pasadena, California 91125, United States

## Abstract

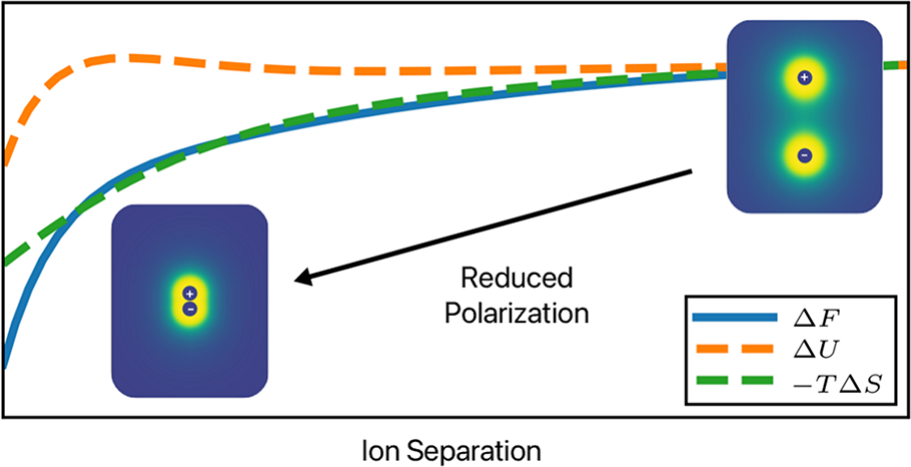

Implicit solvent models that reduce solvent degrees of freedom into effective interaction potentials are widely used in the study of soft materials and biophysical systems. For electrolyte and polyelectrolyte solutions, coarse-graining the solvent degrees of freedom into an effective dielectric constant embeds entropic contributions into the temperature dependence of the dielectric constant. Properly accounting for this electrostatic entropy is essential to discern whether a free energy change is enthalpically or entropically driven. We address the entropic origin of electrostatic interactions in a dipolar solvent and provide a clarified physical picture of the solvent dielectric response. We calculate the potential of mean force (PMF) between two oppositely charged ions in a dipolar solvent using molecular dynamics and dipolar self-consistent field theory. We find with both techniques that the PMF is dominated by the entropy gain from the dipole release, owing to the diminished orientational polarization of the solvent. We also find that the relative contribution of the entropy to the free energy change is nonmonotonic with temperature. We expect that our conclusions are applicable to a broad range of problems involving ionic interactions in polar solvents.

## Introduction

A wide variety of simulation and theoretical approaches utilize implicit solvent models, where the solvent degrees of freedom are lumped into effective interactions.^1,2^ Treating the solvent as a background medium can significantly reduce the computational cost.^3−5^ In doing so, however, the solvent degrees of freedom become hidden in effective interaction potentials, which are often specified in an approximate manner. Common methods in biological simulations include the accessible surface area (ASA) method and continuum electrostatic methods such as the generalized Born model.^6−13^

Dielectric materials with polar molecules respond to an electric field through reorientation of the dipoles.^14^ Generally, the presence of an electric field will cause the dipoles to align and increase the order in the system. The free energy change during dipole reorganization is thus composed of both energetic and entropic contributions.^15^ The energy comes from the electrostatic interactions of the dipoles with the electric field and with each other, while the entropy arises from the changes in the orientation of the dipoles. For a uniform dielectric material, the electrostatic entropy contribution is encapsulated in the temperature dependence of the dielectric constant.^16^
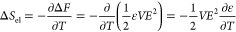
1where *T* is the temperature, Δ*S*_el_ is the electrostatic entropy change due to the application of an electric field, Δ*F* is the Helmholtz free energy change, ε is the dielectric constant, *V* is the system volume, and *E* is the electric field. Capturing the entropic contribution relies on knowing the temperature dependence of the dielectric constant.

In solutions containing ions and charged macromolecules, the presence of charged species generates the electric field that polarizes the solvent.^16^ Recently, this phenomenon has been used to explain the apparent discrepancy between experiments^17−20^ and coarse-grained molecular dynamics simulations^21−24^ in describing the driving force for polyelectrolyte complex coacervation. Chen and Wang showed that the Coulomb potential used in coarse-grained implicit solvent models inherently includes an entropic contribution, which they term the electrostatic entropy.^25^ By correctly accounting for this electrostatic entropy contribution through the temperature dependence of the dielectric constant, they were able to predict entropy driven coacervation from implicit solvent molecular dynamics, in agreement with experimental observations. Further, they rationalized this entropic driving force as arising from the solvent reorganization using the example of two oppositely charged ions forming an ion pair. The entropic contributions to the potential of mean force have also been addressed in other studies which used molecular dynamics^26^ and the extended reference interaction site model (RISM).^27^ However, these studies were restricted to water, which has specific properties and interactions with different types of ions.

The effective interaction potential, or potential of mean force (PMF), between ions in solution is a result of the combined effects of direct ion–ion interactions and interactions of ions with the solvent. At the most basic level, one can assume the solvent has a uniform temperature-dependent dielectric constant. In the Debye approximation,^28^ for the process of bringing two ions from infinity to a distance *r*, we have
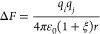
2
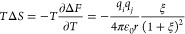
3
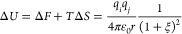
4where *q*_*i*_ and *q*_*j*_ are the charges on the ions, *U* is the internal energy,  is a dimensionless measure of the dipole strength, μ̅ is the dipole moment, β = 1/*k*_B_*T*, and *v* is the molecular volume of the solvent. Thus, the electrostatic entropy will dominate the PMF when ξ > 1. A similar result can be obtained using a more complete dielectric theory, such as Onsager’s theory.^29,30^ While this is a satisfying result, it is phenomenological and does not provide a clear molecular picture, since the solvent degrees of freedom are not explicitly included.

In this study, we address the concept of electrostatic entropy and demonstrate its generality by studying two ions in a dipolar fluid. Using dipolar self-consistent field theory (DSCFT) and molecular dynamics simulations, we analyze the PMF between two oppositely charged, monovalent ions immersed in a dipolar solvent, explicitly accounting for the solvent degrees of freedom. We separate the PMF into its entropic and energetic contributions to determine the conditions where the electrostatic entropy dominates. Finally, we connect the entropic driving force to the release of dipoles as the ions approach one another, quantified through the decrease in the solvent polarization. While solvent reorganization has long been recognized as a source of entropy in physical processes, especially in the context of binding of small molecules to proteins and multivalent ions to charged macromolecules in water,^31−33^ the phenomenon is often presented as solvent- or system-specific, where other effects such as hydrogen bonding may dominate. Our results highlight the generality of the electrostatic entropy and the importance of properly accounting for the solvent degrees of freedom in studying charge-containing systems.

## Methods

### Simulation Model

To model two ions in a dipolar fluid, we use a Stockmayer fluid model based on work by Shock et al.^34^ The solvent particles possess permanent point dipoles **μ** at their center of mass, while the ions are described as point charges with no dipole (Figure S1). The nonelectrostatic nonbonded potential energy for all bead types is described by a truncated and shifted Lennard-Jones (LJ) potential,^35^
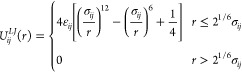
5where ,  for all pairs, and *r* is the distance between beads *i* and *j*. With a cutoff of 2^1/6^σ_*ij*_, the LJ potential is purely repulsive and ϵ_*ij*_ is relatively unimportant. For all of our systems, we set ϵ_*i*_ = ϵ_*j*_ = 1.

The electrostatic interactions are composed of charge–charge, charge–dipole, and dipole–dipole interactions. The standard Coulomb potential describes the charge–charge interactions between the two ions,
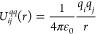
6where ε_0_ is vacuum permittivity and *q* is the charge on each ion. The charge–dipole and dipole–dipole interactions are, respectfully, given as
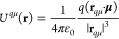
7where |**r**_*q*μ_| is the center of mass distance between the charge and the solvent dipole, and
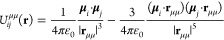
8where |**r**_μμ_| is the center of mass distance between point dipoles **μ**_*i*_ and **μ**_*j*_. We reiterate that only the ions carry charge. All long-range electrostatic interactions are computed using a standard Ewald summation.

The solvent dipoles reorient due to the torque arising from charge–dipole or dipole–dipole interactions. We use a Langevin thermostat that takes into account the angular degrees of freedom of the solvent. The LJ size parameter σ_*s*_ is used to describe the spherical diameter required to update the angular velocity via the solvent bead’s moment of inertia.

Throughout the work, we consider a coarse-grained model solvent based on water.^34^ Namely, the number density is ρ = 0.03344/Å^3^ at *T* = 300 K, with diameter σ_*s*_ = 3.0 Å and mass 18.015 . Water has a gas-phase dipole moment of 1.85 D; however, we vary the magnitude of the dipole moment to highlight the role of the dipoles, ranging from μ̅ = 0–2 D. For simplicity, we treat the anion and cation as monovalent ions ±1*e* with the same size parameter as the solvent, σ_+_ = σ_–_ = σ_*s*_. For all simulations, we use reduced units with the length scale 1σ = 3 Å, energy scale 1ϵ = 2.49 kJ/mol, and mass scale 1*m* = 18.015 g/mol that give a corresponding time scale .

The PMF between two ions is calculated using the adaptive biasing force (ABF) method.^36,37^ The ion separation distance is divided into 8 windows in the range of 0.75σ to 8.5σ. In each window, the system was equilibrated for 5 × 10^6^ timesteps (δ*t* = 0.005τ) and production of 10^7^ timesteps. Each simulation consists of 5000 solvent particles, corresponding to a simulation box length of 17.69σ. We use the GPU^38,39^ and Colvars^40^ packages in lammps(41) for simulations and ovito(42) for visualizations. Example lammps and Colvars scripts for calculating the PMF are available at https://github.com/chrisbalzer/Stockmayer-Two-Ions.

### Field-Theoretic Model

In recent years, several groups have developed statistical field theories that explicitly account for solvent polarization and the dielectric response.^43−47^ Here, we adapt a dipolar self-consistent field theory that was previously developed and used to study ion solvation energy and electron transfer reorganization energy.^48−50^ Alternatively, we could have used the Ornstein–Zernike integral equation theory with the hypernetted-chain (HNC) approximation, or the extended RISM equation which have both been used to study the potential of mean force between ions at infinite dilution.^27,51−54^ The purpose of the theoretical model is not to compare or validate the simulation, but rather to provide an alternative approach for studying the ionic interaction in dipolar fluids to emphasize the generality of the presented behavior. We believe any theory which can capture the solvent orientational polarization in the vicinity of ions can capture the presented results, which includes all of the mentioned theories. We consider two ions at fixed separation immersed in a dipolar solvent. The solvent dipole is composed of a permanent dipole **μ** and an induced dipole ***p***. The induced dipole is related to the electronic degrees of freedom. This contribution is not essential for this work, but we include it for completeness as it does not add much complexity to the theory or calculations. The ions are modeled as Gaussian smeared charges inside a spherical solute cavity, where the solvent is excluded. The use of smeared charges is for convenience, to avoid the diverging self-energy. The cavities are modeled using a spherically symmetric cavity function.^55−57^ The ions are treated as fixed external charges to the solvent in this field theory. See Figure S2 for an example of the system setup. The microscopic charge density in the system is given by the contributions from the two ions and the solvent dipoles.
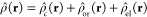
9Here,  is the total charge density of the ions, modeled as Gaussian smeared charges,
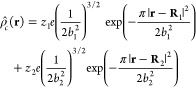
10where *z*_*i*_ are the ion valencies, *b*_*i*_ are the radii of the charge spread, **R**_*i*_ are the ion positions, and *e* is the elementary charge.  and  are the charge densities due to the permanent and induced dipoles of the solvent, respectively. The charge density due to the orientational and electronic contributions of the solvent can be expressed in terms of their dipole moments as
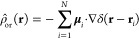
11
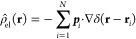
12where *N* is the number of solvent molecules, **μ**_*i*_ is the permanent dipole moment on molecule *i*, and ***p***_*i*_ is the induced dipole moment on molecule *i*. In the energy, we only consider the Coulomb interactions between all charges, and a harmonic penalty associated with the induced dipoles
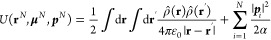
13where α is the polarizability. We work in the grand canonical ensemble with volume *V*, temperature *T*, and solvent chemical potential μ. We assume the system is incompressible. The grand canonical partition function is given by
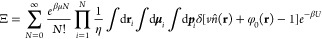
14where *n̂* is the solvent density operator, φ_0_ is the volume fraction occupied by the ions, and η is a factor similar to the cube of the thermal wavelength, which has no thermodynamic consequence. The solvent density operator is given by . The local volume fraction due to the two ions, is modeled by the superposition of two cavity functions,
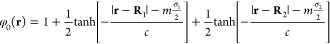
15where σ_*i*_ is the diameter of the two ions, *m* is a positive parameter for shifting the boundary of the cavity, and *c* is a positive parameter for tuning the width of the interface between the ion and the solvent. In practice, any reasonable choice of *m* and *c* will yield the same qualitative results. We choose *c* = 0.01 for a rapid but continuous decay near the ion boundary, and we choose *m* = 0.95 such that the ion volume fraction practically decays to zero by σ_*i*_.

We take advantage of the Fourier representation of a delta functional to introduce the incompressibility field *w*
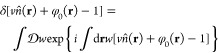
16To turn the partition function into a field theory, we introduce coarse-grained density fields ρ_or_ and ρ_el_ through the identities
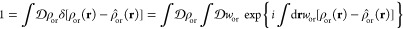
17
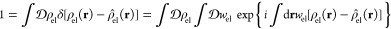
18where *w*_or_ and *w*_el_ are auxiliary fields introduced through the Fourier representation of the delta functional. In eqs 16–18, the notation  denotes functional integration. Applying these identities to the partition function results in
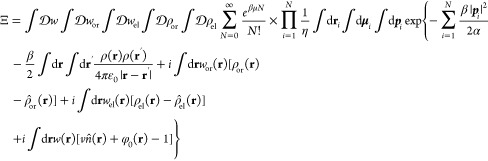
19where . Through these identity transformations, the particle–particle interactions are turned into particles interacting with fluctuating fields. The particle degrees of freedom can be easily integrated out to yield the single-particle partition function, *Q*[*w*, *w*_or_, *w*_el_], given by
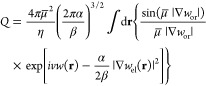
20The transformed grand canonical partition function is now written as
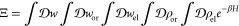
21where the Hamiltonian, *H* = *H*[*w*, *w*_or_, *w*_el_, ρ_or_, ρ_el_], is a functional of the field variables only and is given by
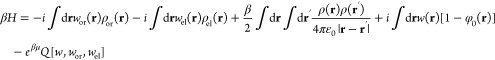
22The integrals in the partition function cannot be evaluated in closed form, so we use the saddle-point configuration as an approximation to the full partition function. The saddle point is found by extremizing the Hamiltonian with respect to all the field variables.^58^ The grand free energy is given by βΩ = – ln Ξ ≈ – ln Ξ*, where Ξ* is the saddle-point contribution to the partition function. Extremizing the free energy results in a set of self-consistent equations that can be solved to find the equilibrium field configurations, and therefore observables such as the free energy and the electric field. Since the saddle-points for the *w* fields lie on the imaginary axis, to avoid the use of imaginary numbers, we define real potentials β*u* = −*iw* and βϕ = *iw*_or_ = *iw*_el_ (*w*_or_ = *w*_el_ at equilibrium). The set of self-consistent equations obtained from extremizing Ω with respect to *w*, ρ_or_ (and ρ_el_), *w*_or_, and *w*_el_ respectively, is given by
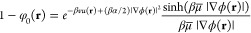
23
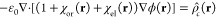
24
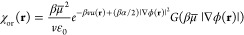
25
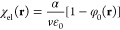
26where *G*(*x*) = [1/ tanh(*x*) – 1/*x*] sinh(*x*)/*x*^2^. Additionally, we have defined the orientational electric susceptibility, χ_or_, and the electronic electric susceptibility, χ_el_. For convenience, The Poisson equation, eq 24, is written in a way that separates the ion charge density from the bound solvent charge density (reflected in χ_or_ and χ_el_). Equation 24 can be rewritten as
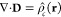
27where **D** = −ε_0_[1 + χ_or_(**r**) + χ_el_(**r**)]∇ϕ(**r**) is the electric displacement. Conveniently, the electric displacement can be determined solely from the location of the free charges. We simplify the solution to eq 27 by assuming that the total electric displacement is the superposition of the electric displacement due to each of the ions. (The superposition principle is used as an approximation to avoid solving the full Poisson equation. In general it does not hold exactly for nonlinear dielectrics. The approximation becomes less accurate for strong interactions, and in particular when the ions are at close approach.^49^) The displacement at a distance *r*_*i*_ from an isolated Gaussian smeared charge *i* is
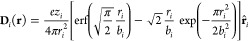
28where **r**_*i*_ = **r** – **R**_*i*_, and  is the unit vector in the direction of **r**_*i*_. The equilibrium electric field is found by iterating **D**(**r**) = −ε(**r**)∇ϕ(**r**) using eqs 23, 25, and 26 to calculate the dielectric function ε(**r**) = ε_0_[1 + χ_or_(**r**) + χ_el_(**r**)]. For all self-consistent calculations, we iterate until the maximum difference in the electric field between two consecutive steps is less than 10^–13^.

The equilibrium free energy can be simplified by plugging the saddle-point equations into the Hamiltonian,
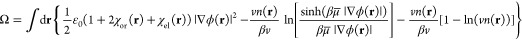
29where *vn*(**r**) = 1 – φ_0_(**r**) is the coarse-grained solvent volume fraction. The system is rotationally symmetric about the axis connecting the ions, allowing the integral in eq 29 to be calculated in cylindrical coordinates with variation restricted to the *r*, *z*-plane. We use a large enough domain to avoid cutting off the long-range tail of the electric field from the ions.

When calculating the PMF, the quantity of interest is the difference between the free energy at separation *r* and that at infinite separation. We call this difference ΔΩ = Ω(*r*) – Ω(*∞*). The entropic contribution to ΔΩ can be calculated with the derivative
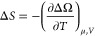
30Note that the change in grand free energy is equal to the change in Helmholtz free energy for this process as the chemical potential and particle number do not change when bringing the ions together at a fixed *T* and *V* due to the incompressibility constraint. Mathematically, we can write the following
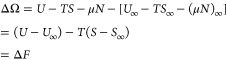
31where the subscript *∞* denotes a quantity at infinite ion separation. We then decompose ΔΩ into entropic and energetic contributions by calculating Δ*S* with eq 30 and internal energy with Δ*U* = Δ*F* + *T*Δ*S* = ΔΩ + *T*Δ*S*.

## Results and Discussion

**Figure 1:**
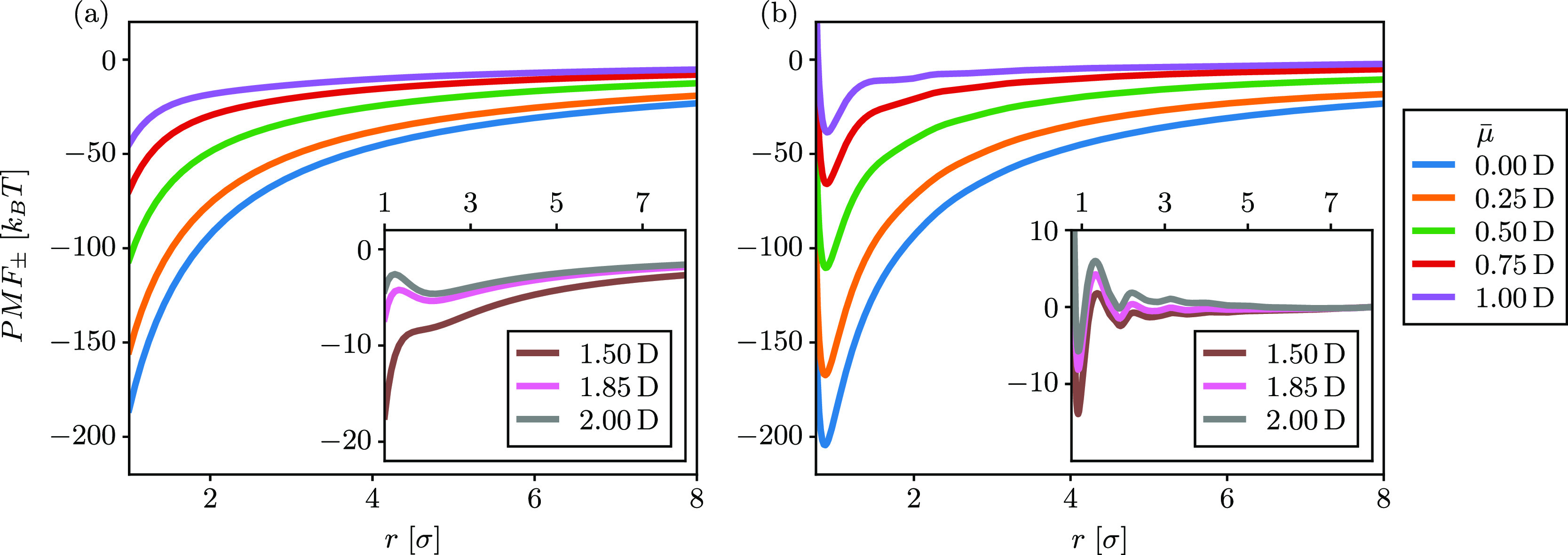
PMFs for various dipole moments calculated from (a) DSCFT and (b) molecular dynamics simulation with σ = 3 Å, *T* = 300 K, *q*_1_ = −*q*_2_ = *e*, *v* = 30 Å^3^. The insets of both panels show PMFs for larger dipole moments. For reference, μ̅ = 1.85D corresponds to the gas-phase dipole moment of water.

We start with a discussion of the behavior of the PMF for bringing two oppositely charged monovalent ions together in a dipolar solvent; this is shown in Figure 1. Both panels show that increasing the dipole moment of the solvent decreases the attraction between the two ions. This behavior can be easily understood as due to the increase in the effective dielectric constant with the dipole moment. The diverging behavior below *r* = 1σ in Figure 1b is due to the diverging LJ potential from simulation. Above *r* = 1σ, we see close agreement between the DSCFT and simulation results at low to moderate dipole moment; a direct comparison is given in Figure S3 in the Supporting Information. At higher dipole moments (insets of Figure 1), oscillations appear in the PMFs for simulations, indicating that there are strong solvation shells of the dipolar solvent around the ions, with each maximum corresponding to the energy barrier for breaking a solvation shell.^59^ Interestingly, for large dipole moments, even DSCFT captures (though to a lesser degree) this nonmonotonic behavior, despite it only accounting for the solvent excluded volume through the incompressibility constraint without explicitly considering the packing of the solvent molecules.

For large ion separations, the PMF from both DSCFT and simulation reproduces the expected Coulomb behavior, Δ*F*(*r*) = −*e*^2^/4πε*r*, where ε is the effective dielectric constant for the given dipole moment. Table S1 gives the effective dielectric constants obtained by fitting the long-range part of the PMF to a Coulomb potential. The dielectric constants so obtained from the simulation are consistent with literature values for small dipole moment.^60−63^ The effective bulk dielectric constant from DSCFT is equivalent to that of Debye^28,47^. The effective bulk dielectric constant from MD simulations is higher than from DSCFT, since the simulations inherently include the reaction field due to dipole–dipole correlations.^29,62^ It is possible to include the reaction field effect in the field theory, as was done by Zhuang and Wang;^47,64^ however, this would add significant complexity and would not affect the qualitative behavior observed in this study.

**Figure 2:**
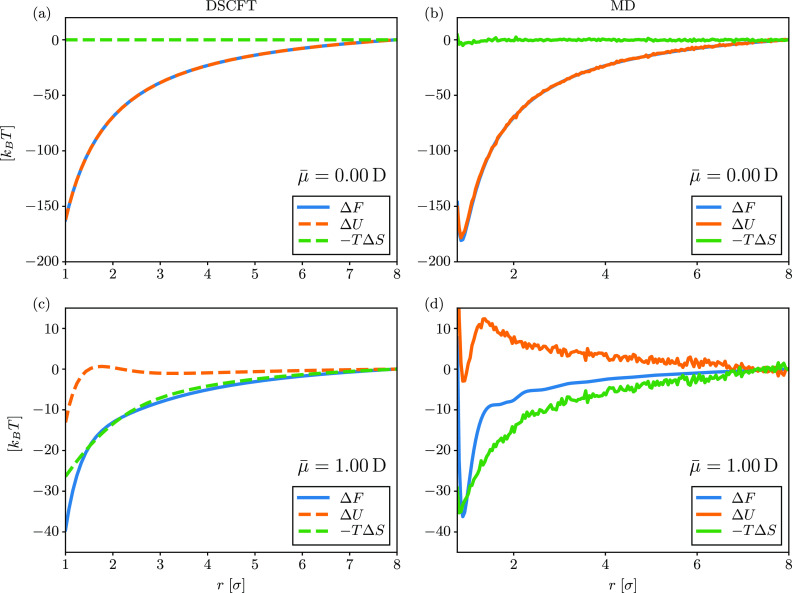
PMFs decomposed into their energetic and entropic contributions with solvent dipoles of (a, b) μ̅ = 0 D and (c, d) μ̅ = 1 D. The PMFs are calculated from DSCFT in (a) and (c) and MD simulation in (b) and (d). Other parameters are the same as in Figure 1.

We decompose the PMF into its energetic and entropic contributions in Figure 2. From the MD simulation, the entropic contribution is calculated using – *T*Δ*S* = Δ*F* – Δ*U*, where Δ*U* is the potential energy calculated from the pair potentials (the kinetic energy is constant at any given temperature and thus does not contribute to the energy change). From DSCFT, the entropy is calculated via eq 30. Since it is difficult to calculate these differences with respect to infinite separation in simulation, we choose a reference point of *r* = 8σ (the largest simulated separation). The DSCFT is not limited by this constraint but we use the same reference point for consistency.

In Figure 2a,b, we see that there is essentially no entropic contribution to the PMF when there is no solvent dipole, indicating that the PMF is only made up of the electrostatic interaction energy. When the solvent does have a permanent dipole, as in Figure 2c,d, we see that the PMF is dominated by the entropic contribution. The energetic contribution even becomes unfavorable, which was similarly observed by Chen and Wang.^25^ The shape and magnitude of the free energy, internal energy, and entropy are similar to those observed in previous simulation and theoretical studies that focused on ions in water.^26,27^ As we discuss later, this significant entropy increase comes from the diminished polarization of the solvent around the ions. The unfavorable energetic contribution comes from the fact that ion–dipole interactions are weakened when the ions are close. Physically, the two ions form an effective dipole upon close-contact, and dipole–dipole interactions are weaker than ion–dipole interactions. We provide similar plots for additional values for the dipole moment in Figure S4 in the Supporting Information.

**Figure 3:**
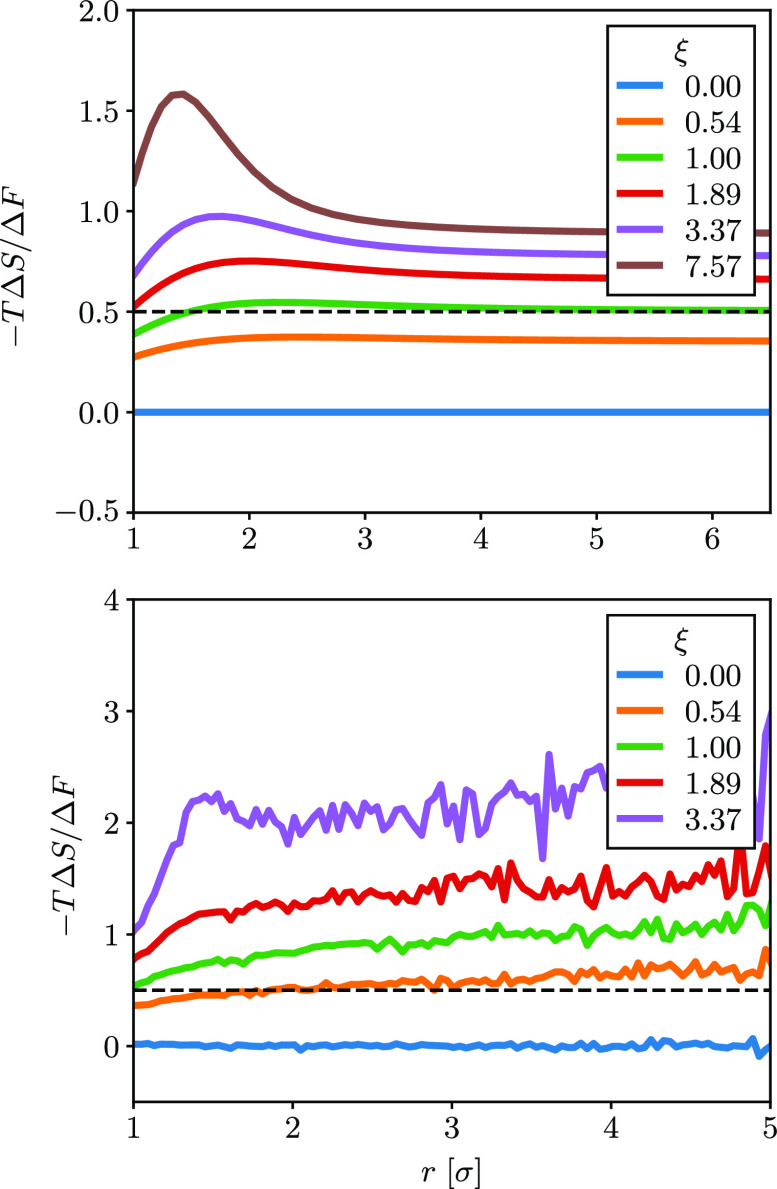
Ratio of PMF due to entropy for σ = 3 Å, *q*_1_ = −*q*_2_ = *e* and various  calculated via (a) DSCFT and (b) molecular dynamics.

For clarity, we explicitly calculate the entropic contribution as a ratio to the total free energy difference in Figure 3 for different values of the dimensionless dipole parameter ξ. For ξ = 1, the entropic and energetic contributions are equal at long-range for DSCFT. This is exactly what is predicted when using the Debye approximation for the dielectric constant in the Coulomb potential, as shown in eqs 2–4. Similarly, the MD simulations predict a crossover from energetically to entropically dominated, but the crossover is actually below ξ = 1 because of the stronger dipole–dipole correlations discussed earlier. However, determining the exact crossover from simulation is computationally demanding owing to both the required system size and sampling of the potential energy, particularly at large ξ, so we mostly focus on the qualitative features. Importantly, Figure 3 shows that the crossover from energy to entropy driven is obtained by increasing the dipole moment or decreasing the temperature.

**Figure 4:**
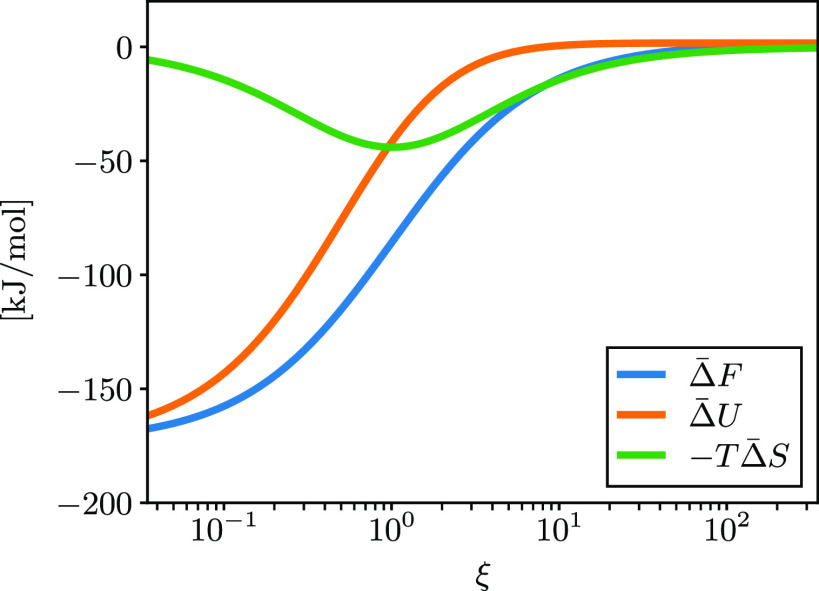
Free energy, internal energy, and entropy change from infinite separation to *r* = 5σ vs  for σ = 3 Å and *q*_1_ = −*q*_2_ = *e*. Calculations were done using DSCFT.

In the Debye theory, ξ is a universal parameter that quantifies the importance of polarization in the system. It is of interest to see how the electrostatic entropy contribution changes with this parameter. To this end, we consider the various components of the PMF in the DSCFT at a fixed distance *r* = 5σ, where the symbol Δ denotes the difference from the infinite separation. The result is shown in Figure 4. We see that for low ξ (weak dipoles or high temperature) the entropy change is small. Near ξ = 1 there is the crossover from energy to entropy dominance, consistent with the Debye analysis and the results of Figure 3. In the high ξ regime (strong dipoles or low temperature), the process is practically fully entropy driven. The free energy and entropy change are small in the high ξ regime due to the large effective dielectric constant. At the mean-field level, the same curves are observed regardless of whether the temperature or solvent dipole is changed, indicating that ξ is a universal parameter for determining the entropic contribution to the free energy. This holds true as long as the ions are not too close together.

**Figure 5:**
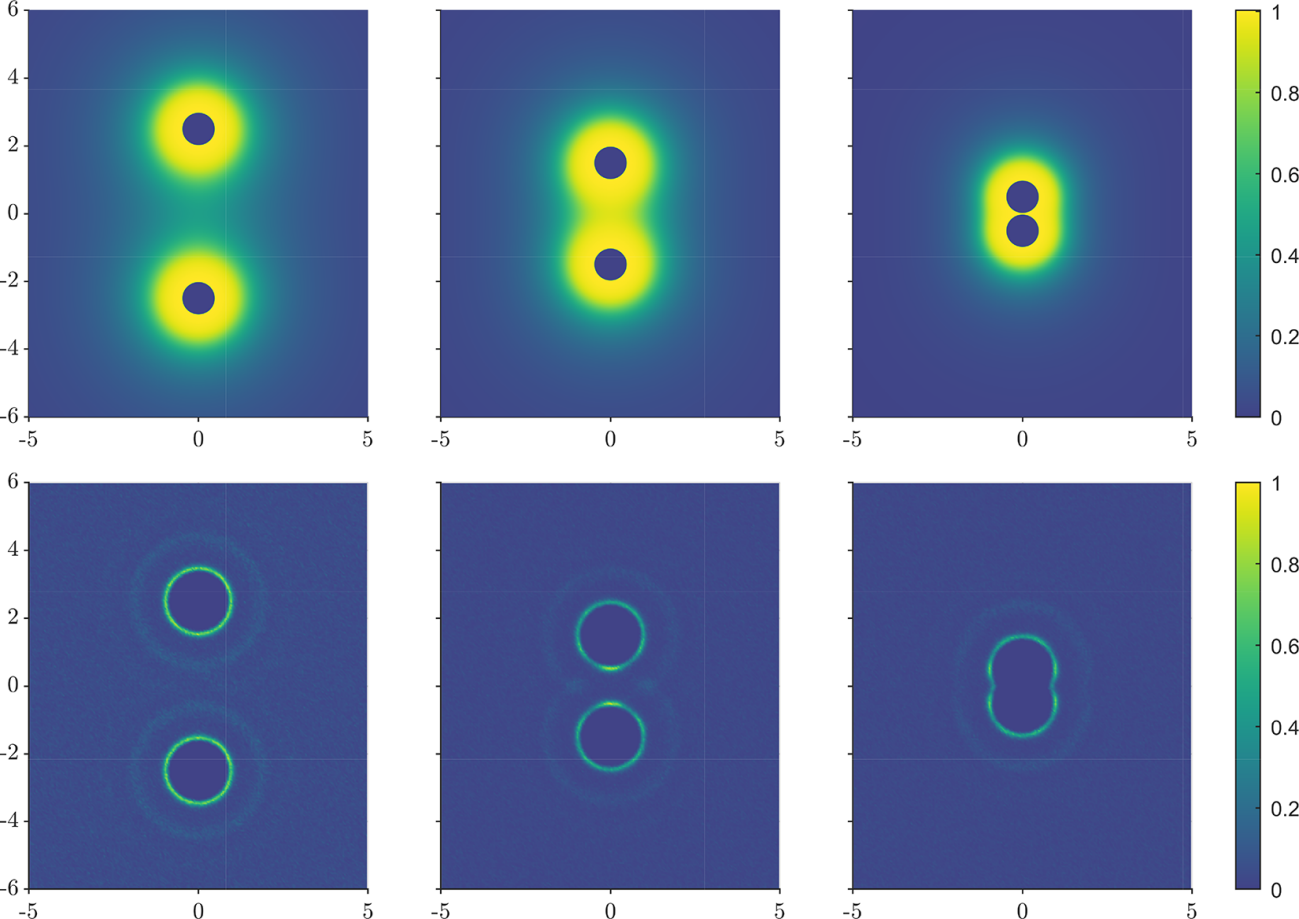
Solvent polarization at the midplane of the ions for both theory (top row) and simulation (bottom row). Spatial positions are in units of σ. The ions are at separations of *r* = 5σ, 3σ, and 1σ going from left to right. Both ions have size σ = 3 Å and charges *q*_1_ = −*q*_2_ = *e*.

To visualize the decrease in the orientational order when the two oppositely charged ions approach each other, Figure 5 shows a heatmap of the local polarization induced by oppositely charged ions from both theory and simulation. One could also plot the dielectric function, ε_*r*_(**r**), as was done in previous work using lattice Monte Carlo simulations.^65^ We have provided an example of this type of plot calculated using the DSCFT (see Figure S5). The conclusions drawn from both types of plots are the same; however, we believe the plot of local polarization provides a clearer picture of solvent release. In DSCFT the measure of local orientational polarization is taken to be the orientational susceptibility times the electric field, or |*P*| = χ_or_|∇ϕ|. Note that far away from the ions, the orientational polarization is zero due to the random orientation of the dipoles. In the simulation, the measure is given by the magnitude of the local, time-averaged solvent dipole, |⟨**μ**(**r**)⟩| (simulation details are in the Supporting Information). Both rows of Figure 5 are normalized by each row’s maximum value to emphasize the qualitative features. The qualitative difference between the simulation and theory comes from the strong ordering and solvation shells present in the simulation, of which the first two are clearly visible in Figure 5b. This strong spatial ordering is not captured in the DSCFT, and therefore the polarization is continuous and decreases with distances away from the ions. The shrinking of the cloud surrounding the ions indicates that the polarization around the ions weakens as the ions move closer together. The total polarization due to two separated ions is greater than that of the paired ions, meaning that a portion of the solvent is released to freely translate and rotate upon bringing the ions together. This solvent release is responsible for the large entropy increase, which dominates the ion interactions at a sufficiently large dipole moment or low temperature.

**Figure 6:**
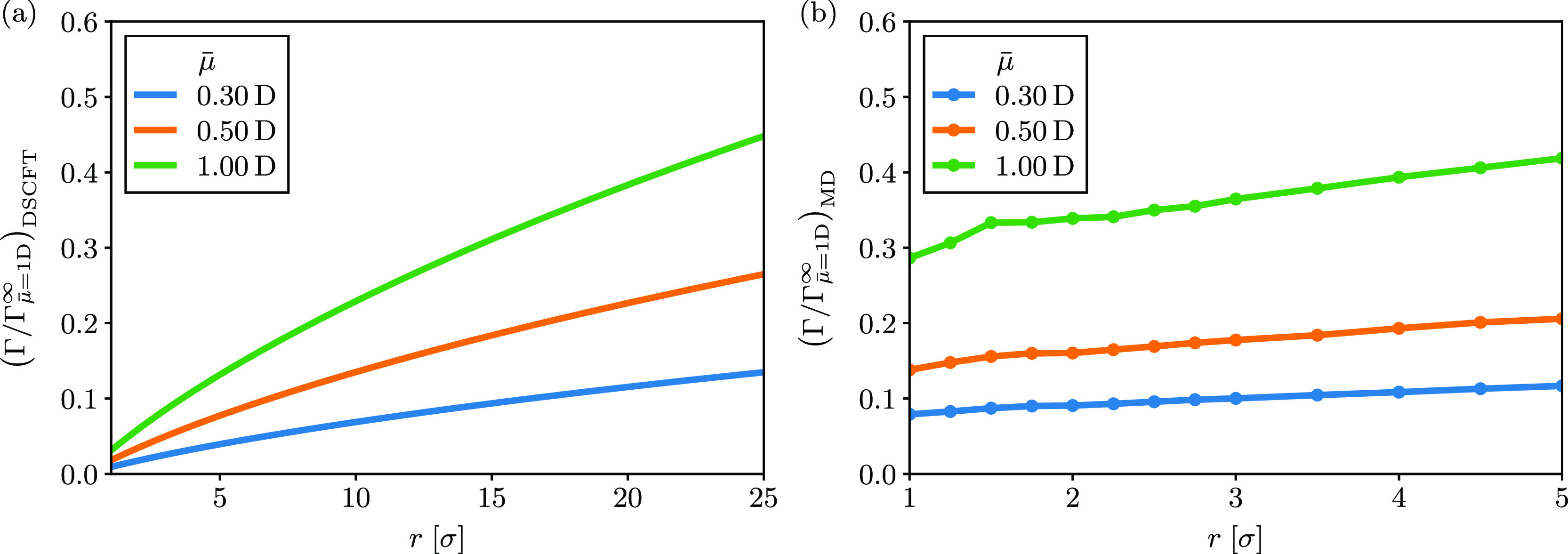
Normalized excess polarization versus the ion separation for various dipole moments μ̅, near the energy/entropy crossover, with σ = 3 Å and *q*_1_ = −*q*_2_ = *e*. Here, the excess polarization is normalized by the infinite separation excess polarization for μ̅ = 1.0 D. Calculations were done using (a) DSCFT and (b) simulation.

While the visual representation is helpful it does not provide a quantitative measure of the change in polarization. Therefore, in Figure 6 we quantify the change in excess polarization of the solvent when bringing the ions together. The excess polarization calculated from DSCFT is defined as Γ_DSCFT_ = ∫d**r**χ_or_(**r**)|∇ϕ(**r**)|, which is the local orientational polarization of the solvent integrated over the system volume. From MD simulations, we measure the excess polarization by integrating the time-averaged dipole moment with the local solvent density, Γ_MD_ = ∫d**r**|⟨**μ**(**r**)⟩|ρ_s_(**r**). To highlight the dependence of the magnitude of the excess on the dipole moment, we normalize each panel by the same value. We opt to use the excess polarization at infinite separation from the largest dipole moment, μ̅ = 1 D.

From theory and simulation, the excess polarization decreases as the ion separation decreases. The magnitude of the decrease becomes more substantial with increasing dipole strength. This supports the idea that the solvent dipoles are released as the ions come together, which is responsible for the significant increase in the system entropy. We see that the solvent reorganization is significant even at large separations, where the DSCFT has not plateaued even at *r* = 25σ. Interestingly, in DSCFT, the curves at different dipole moments collapse onto a single curve when each curve is normalized by its excess polarization at infinite separation (see Figure S6). The collapse of the curves indicates that the magnitude of the decrease in solvent polarization is what causes the crossover from energy- to entropy-driven, rather than a new physical process.

## Conclusion

For two monovalent ions in a dipolar fluid, the solvent plays a major role in the interaction of the ions. At the mean-field level, the interaction is entropy dominated when , as is the case of water at room temperature. The physical origin of the entropy is the reorganization of solvent dipoles as the two ions approach one another. This entropy is purely electrostatic in nature, arising from the polarization due to the electric field generated by the ions. We emphasize that this entropy and the solvent reorganization will occur in any polar solvent. In implicit solvent models, the solvent contribution to the entropy may be buried in effective interaction parameters, requiring careful treatment to calculate the entropy of the system. For weakly or moderately charged systems, using the temperature dependence of the bulk dielectric constant may be sufficient to extract the entropic contribution. Such an approach will likely become inadequate in systems with stronger correlations^66^ (i.e., packing effects, multivalent ions, polyelectrolytes, etc.) or nonaqueous solvents,^67^ where explicitly considering the solvent becomes necessary. Even in the model systems considered in this work, for large dipole strengths, the effective interaction (PMF) can no longer be described as a simple Coulombic potential with an effective dielectric constant at short distances.

The implications of these conclusions are important since assembly in charged systems is ubiquitous.^68−71^ For instance, in drug discovery, researchers often discuss enthalpy–entropy compensation, where a strong enthalpic interaction between two species (i.e., protein–ligand) invariably comes with an equivalent entropic compensation. As reemphasized by Dragan et al.,^72^ a significant part of this entropy likely comes from the solvent release. Emphasizing solvation rather than high-affinity ligands could lead to new design strategies in drug discovery. On the simulation side, implicit solvent models are growing increasingly complex, including many machine-learned models.^73^ These models are excellent in their ability to reproduce thermodynamic quantities like the PMF. We hope that our study motivates future efforts toward understanding the components of the PMF, such as the entropic vs energetic contributions, and the specific molecular mechanisms that are responsible for these contributions.

## Supporting Information Available

Simulation and theory system setup. Direct comparison of PMFs for DSCFT and MD simulations. Additional data for entropy/energy. Additional simulation details. Example of dielectric function calculated via DSCFT. Excess polarization normalized for each dipole moment (PDF)
