# Supplementary material for: Entropic Origin of Ionic Interactions in Polar Solvents
Source: J Phys Chem B. 2023 May 9;127(19):4328–37. doi: 10.1021/acs.jpcb.3c00588 (PMC10201535; doi:10.1021/acs.jpcb.3c00588)
Supplement: Supplementary file 1 — jp3c00588_si_001.pdf [file jp3c00588_si_001.pdf]

Supporting Information: Entropic Origin of Ionic Interactions in Polar Solvents

Samuel Varner,[†,‡] Christopher Balzer,[†,‡] and Zhen-Gang Wang[*,†]

†*Division of Chemistry and Chemical Engineering, California Institute of Technology, Pasadena, California 91125*

‡*These authors contributed equally.*

E-mail: zgw@caltech.edu

Contents

List of Figures S-2

List of Tables S-2

S1 System Setup S-3

S2 Comparison of PMFs for DSCFT and MD Simulations S-4

S3 Additional Plots of Entropy versus Energy Contributions S-5

S4 Simulation Details for Fixed Ion Separation S-5

S5 Example Plots of Dielectric Function from DSCFT S-6

S6 Additional Plots of Normalized Excess Polarization S-7

List of Figures

S1 Example simulation configuration of two ions in Stockmayer fluid S-3

S2 Example DSCFT system setup S-3

S3 Comparison of PMFs for DSCFT and MD Simulations S-4

S4 PMFs decomposed into energy and entropy S-5

S5 Dielectric Function from DSCFT S-6

S6 Normalized Excess Polarization S-7

List of Tables

S1 Dielectric constants from DSCFT and MD S-4

S1 System Setup

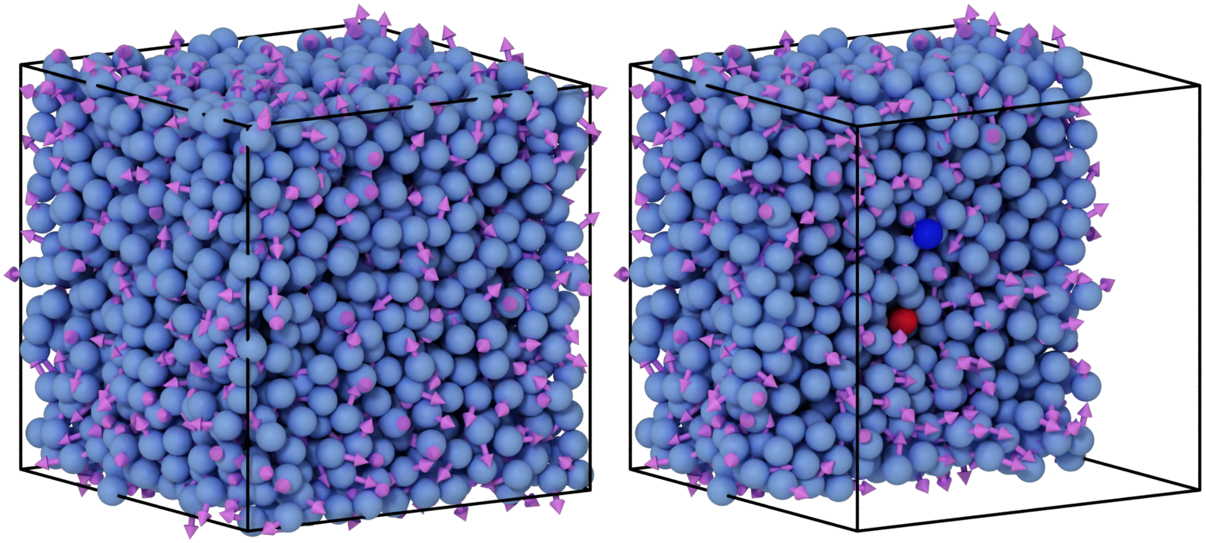

Figure S1: Example simulation configuration of two ions (blue/red) in a Stockmayer fluid (light blue). Solvent dipoles are depicted as pink arrows.

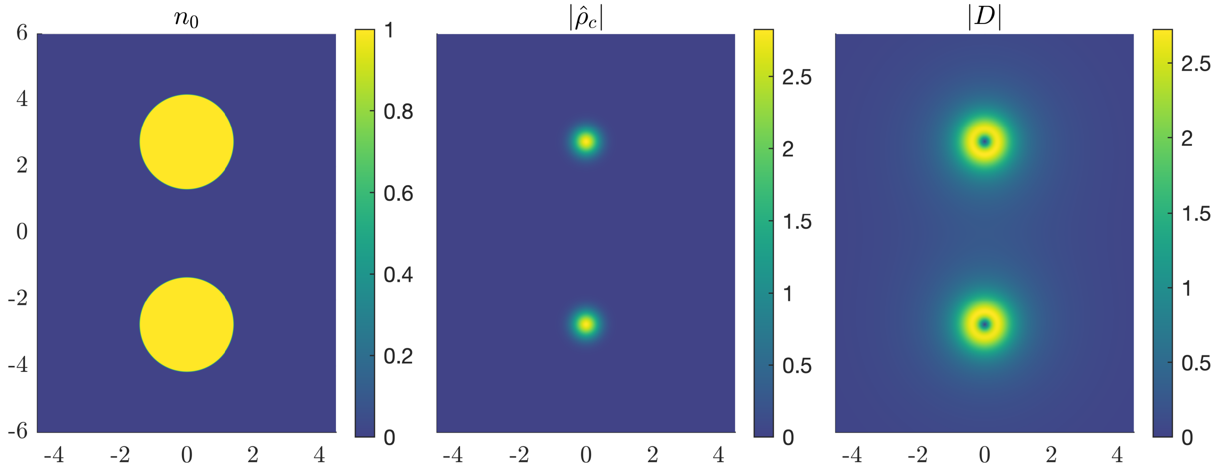

Figure S2: Example plot of ion mass density (left), ion charge density (middle) and electric displacement (right). The plots represent a slice in the x,z-plane. Note that the simulated domain is cylindrical and rotationally symmetric around the axis connecting the ions.

S2 Comparison of PMFs for DSCFT and MD Simulations

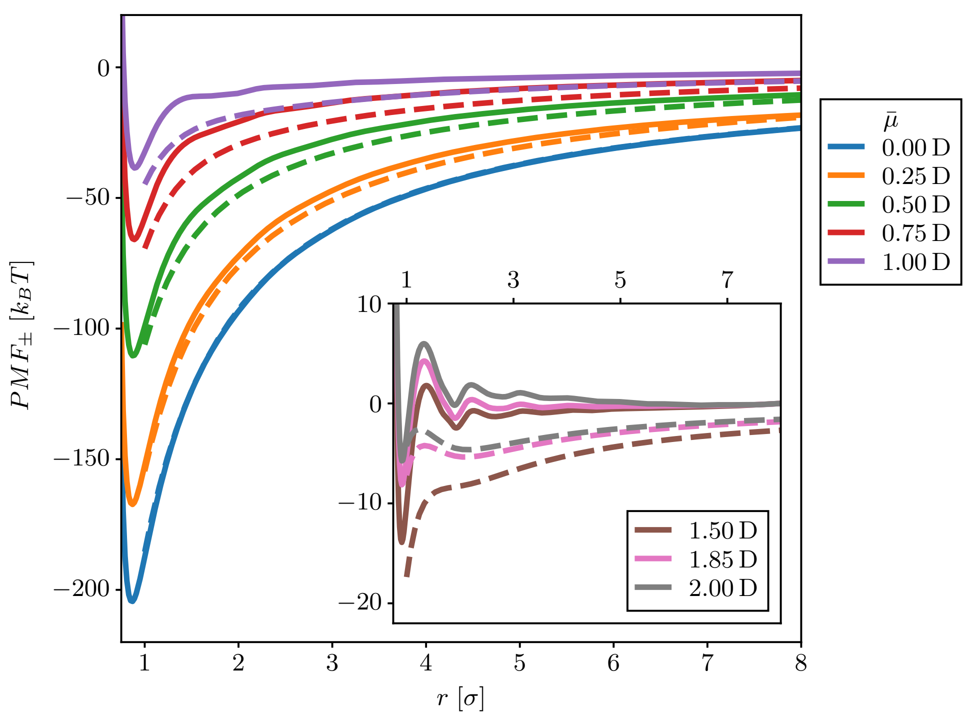

Figure S3: PMFs for various dipole moments with $\sigma = 3\,\text{Å}$, $T = 300\,\text{K}$, $q_1 = -q_2 = e$ and $v = 30\,\text{Å}^3$. The solid and dashed lines are results from MD and DSCFT, respectively. The inset zooms in on higher dipole moments to highlight the strong oscillations.

Table S1: Dielectric constants extracted from fitting long-range PMFs to Coulomb potential. From MD, extracting the dielectric constant from the PMF above 1D is not reliable due to the small oscillations in the PMF arising from packing effects. Other parameters are $\sigma = 3\,\text{Å}$, $T = 300\,\text{K}$, $q_1 = -q_2 = e$, $v = 30\,\text{Å}^3$.

Dipole Moment (D)	ε_r (DSCFT)	ε_r (MD)
0.00	1.00	1.00
0.25	1.21	1.32
0.40	1.54	2.25
0.50	1.84	2.54
0.75	2.89	4.55
1.00	4.37	9.62
1.50	8.57	–
1.85	12.52	–
2.00	14.46	–

S3 Additional Plots of Entropy versus Energy Contributions

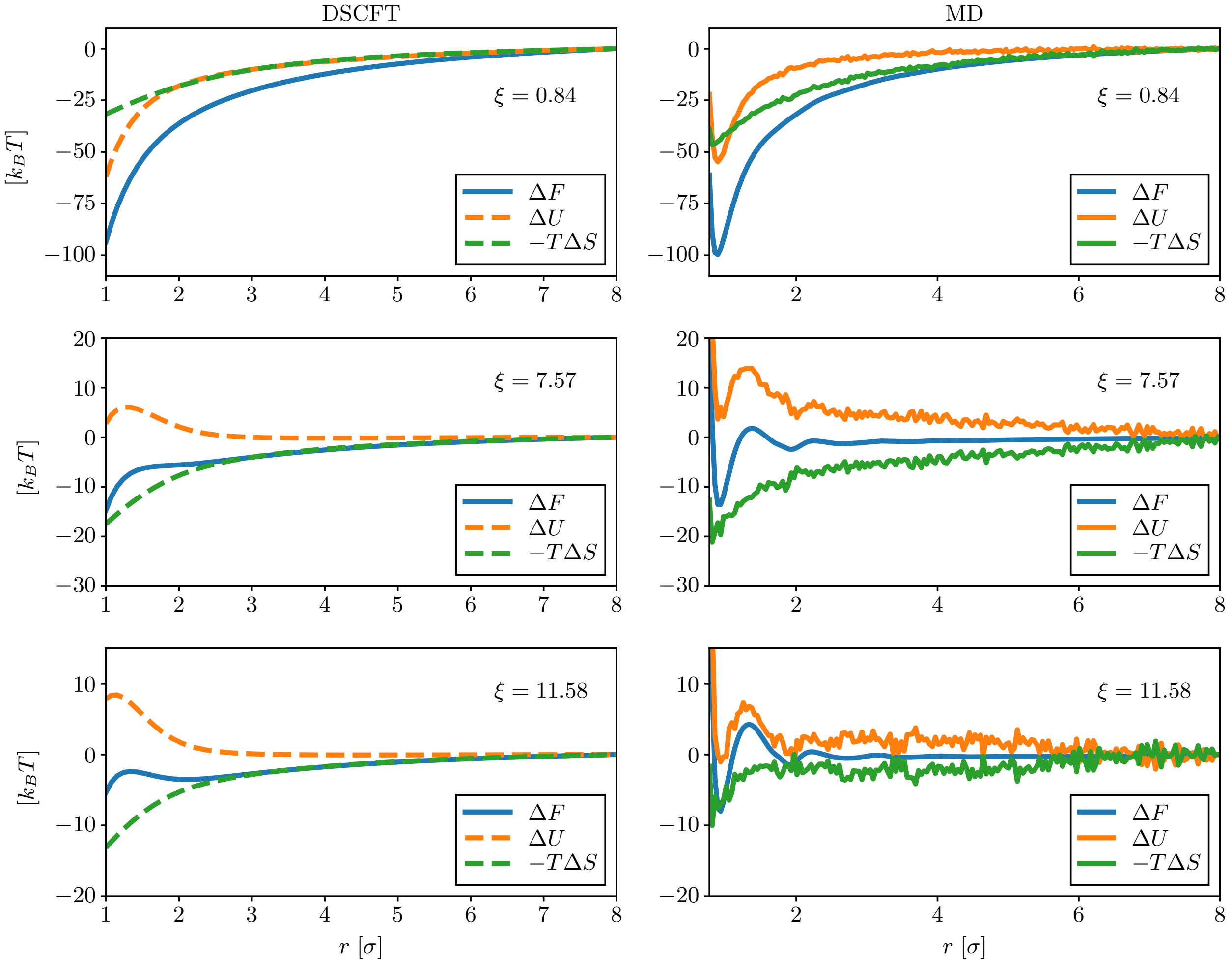

Figure S4: PMFs decomposed into their energetic and entropic contributions for $\sigma = 3\,\text{Å}$, $T = 300\,\text{K}$, $q_1 = -q_2 = e$, $v = 30\,\text{Å}^3$ and various $\xi = \frac{\beta \bar{\mu}^2}{3 v \varepsilon_0}$. The PMFs on the left are calculated via DSCFT and on the right via MD.

S4 Simulation Details for Fixed Ion Separation

To analyze the solvent orientations around two ions at a given separation, we use a slightly different simulation routine from that for calculating the PMFs. For a fixed ion separation, the ion position does not update with each timestep. To accomplish this, the thermostat is only applied to the solvent particles. Each simulation consists of 4000 solvent particles

and two ions of opposite charge with parameters described in the main text. The simulation box size is $15\sigma \times 15\sigma \times 19.69\sigma$ with the longest dimension in the direction of the ion separation. Each system was equilibrated for 5×10^6 timesteps ($\delta t = 0.005\tau$) and production of 10^7 timesteps. To calculate $|\langle \boldsymbol{\mu}(\mathbf{r}) \rangle|$, space is discretized into voxels in which x-,y-,and z-components of the dipole moment are sampled and averaged every 500 timesteps during the production run.

S5 Example Plots of Dielectric Function from DSCFT

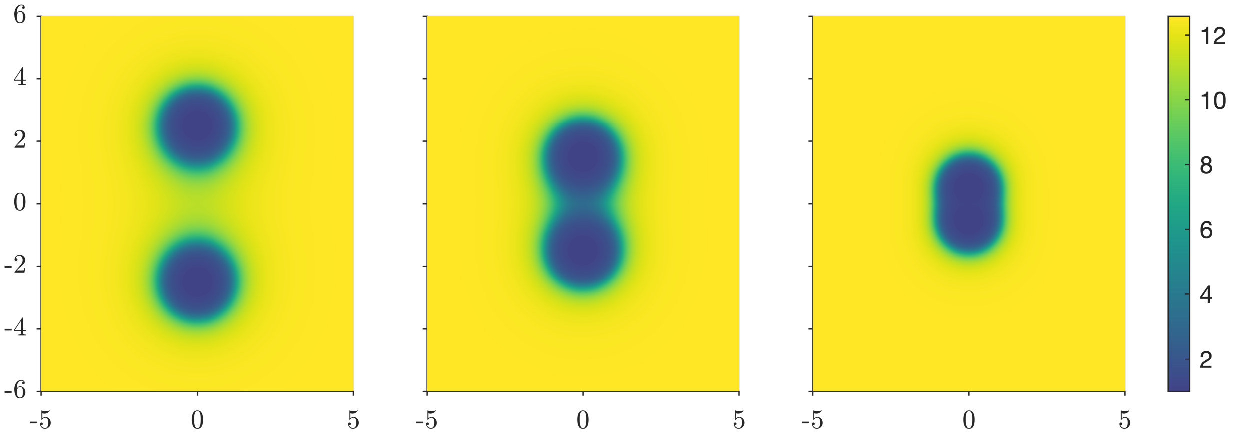

Figure S5: Dielectric function, $\varepsilon_r(\mathbf{r})$ at the midplane of the ions, calculated from DSCFT. Spatial positions are in units of σ. The solvent has the gas-phase dipole moment of water, $\bar{\mu} = 1.85\,\mathrm{D}$. The ions are at separations of $r = 5\sigma$, 3σ and 1σ going from left to right. Both ions have size $\sigma = 3\,\text{Å}$ and charges $q_1 = -q_2 = e$.

S6 Additional Plots of Normalized Excess Polarization

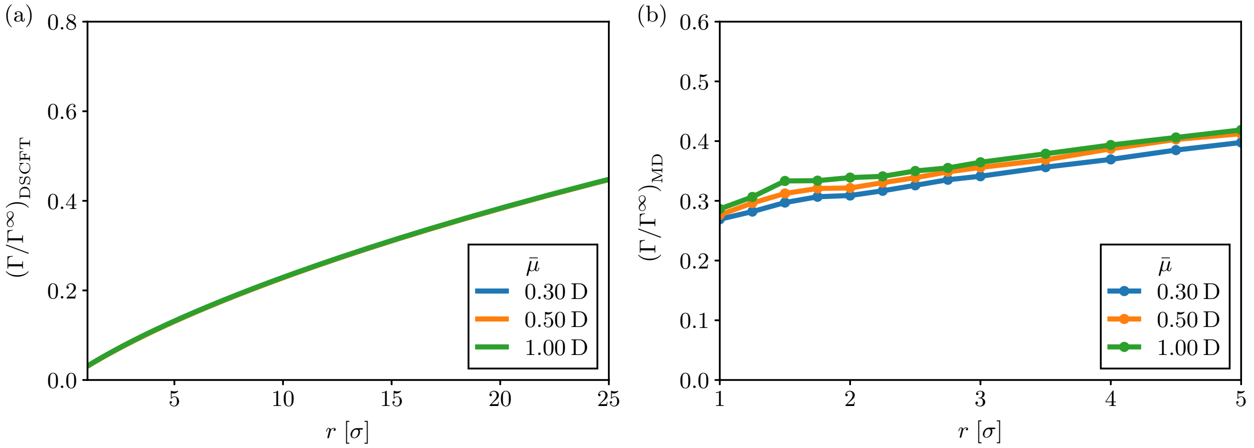

Figure S6: Normalized excess polarization versus the ion separation for various dipole moments $\bar{\mu}$ with $\sigma = 3\,\text{Å}$ and $q_1 = -q_2 = e$. Here, the excess polarization is normalized by the infinite separation excess polarization for each dipole moment. Calculations were done using (a) DSCFT and (b) simulation. We note that when normalized in this way the curves tend to collapse together onto a single curve for DSCFT.
